# Supplementary material for: Synthesis and Evaluation of Clinically Translatable Targeted Microbubbles Using a Microfluidic Device for In Vivo Ultrasound Molecular Imaging
Source: Int J Mol Sci. 2023 May 20;24(10):9048. doi: 10.3390/ijms24109048 (PMC10219500; doi:10.3390/ijms24109048)
Supplement: Supplementary file 1 [file ijms-24-09048-s001.zip › ijms-2344839-supplementary.pdf]

Supplemental information

Figure S1

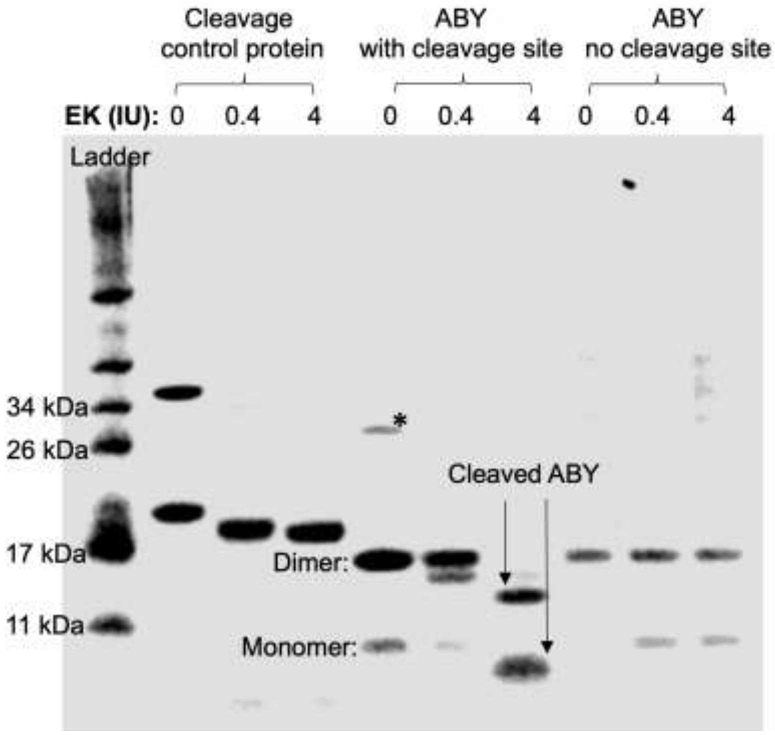

Figure S1. His-Tag removal from ABY ligand to enable its clinical translation. SDS-PAGE analysis of 10 µg AC12 protein treated overnight with enterokinase (EK; 0.4 or 4 IU) to remove the N-terminal His-Tag from the ABY (~8.3 kDa). EK (4 IU) completely cleaved (arrows) AC12 ABY (both monomer ~ 6.9 kDa and dimer ~13.8 kDa) expressed from plasmid with proteolytic recognition sequence (Asp-Asp-Asp-Asp-Lys) placed between the N-terminal His-Tag and the ABY sequences. EK proteolysis-associated protein bands were observed with the positive control protein but not with ABY expressed without the EK recognition site. High amounts of ABY dimer formation in the cleavage assay are observed due to overnight incubation of the non-reduced ABY form to allow optimal EK-mediated cleavage activity.

Figure S2

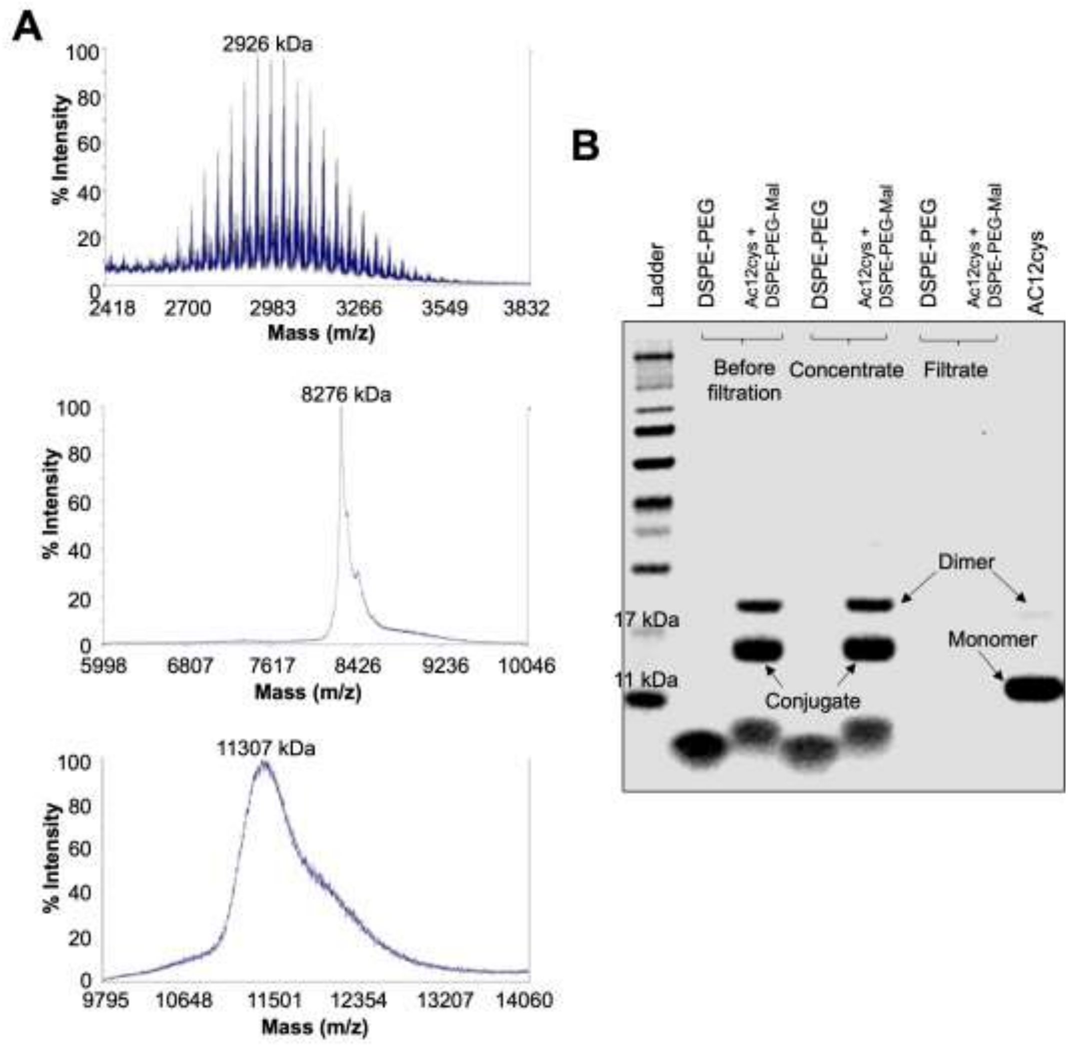

Figure S2. MALDI-TOF analysis and purification of DSPE-PEG-ABY bioconjugate mixture. (**A**) MALDI-TOF analysis of solutions containing DSPE-PEG-Mal phospholipid (top) and AC12cys (middle) in separate solutions or in conjugation mixture (DSPE-PEG-ABY; bottom). m/z peak for the conjugation sample (11307 Da) confirms bioconjugation of AC12cys (Peak m/z: 8276 Da) with DSPE-PEG-Mal (peak m/z: 2926 Da). (**B**) SDS-PAGE analysis of the bioconjugation reaction mixture purified and concentrated through centrifugal membranes with ~3

kDa molecular weight size cut-offs to remove small impurities before the use of bioconjugate mixture in targeted MB production. No sample loss is observed during this step. DSPE-PEG and AC12cys ABY samples are used for reference.

Figure S3

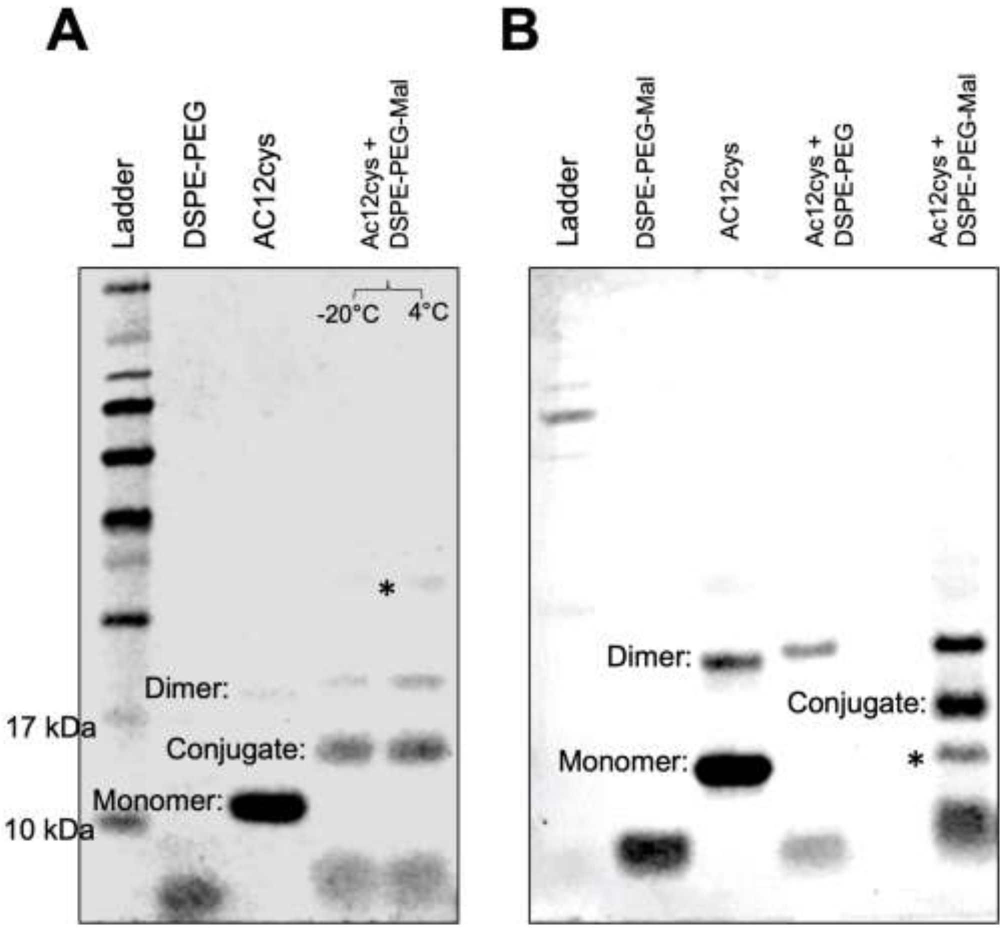

Figure S3. Stability of DSPE-PEG-ABY bioconjugate under various storage conditions. (**A**) SDS-PAGE analysis of bioconjugate sample mixture consisting of AC12cys and DSPE-PEG-Mal stored in freeze-dried form at -20º C for 3 weeks or in water at 4º C for 2 weeks. No bioconjugate degradation is observed in both storage conditions, but a high molecular-weight band, suggesting non-specific aggregation (*), is observed in sample stored at 4º C. (**B**) SDS-PAGE analysis of bioconjugate mixture stored in Lamelli buffer (containing SDS and reactive primary amines from Tris) at 4º C for 3 weeks. Partial degradation (*) of the bioconjugate product is observed under this sub-optimal storage condition.

Figure S4.

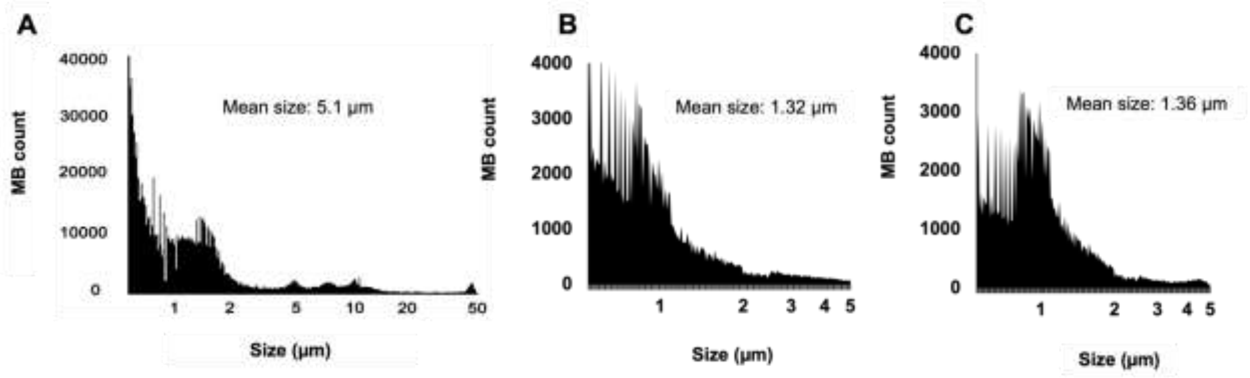

Figure S4: Measurement of MBs size prepared by the vial mixing and microfluidic methods.

MBs were prepared by two (vialmix and microfluidic) approaches and compared the bubbles count and size using acoustic spectroscopy for particle size measurement. Panel A: MBs prepared by vial mix; panel B and panel C: Non-targeted and Targeted MBs prepared by Horizon microfluidic system. Acoustic spectroscopy clearly shown that MBs prepared by Horizon microfluidic system resulted with MBs of uniform and narrow size distribution compared to the vial mix based preparation.

Figure S5

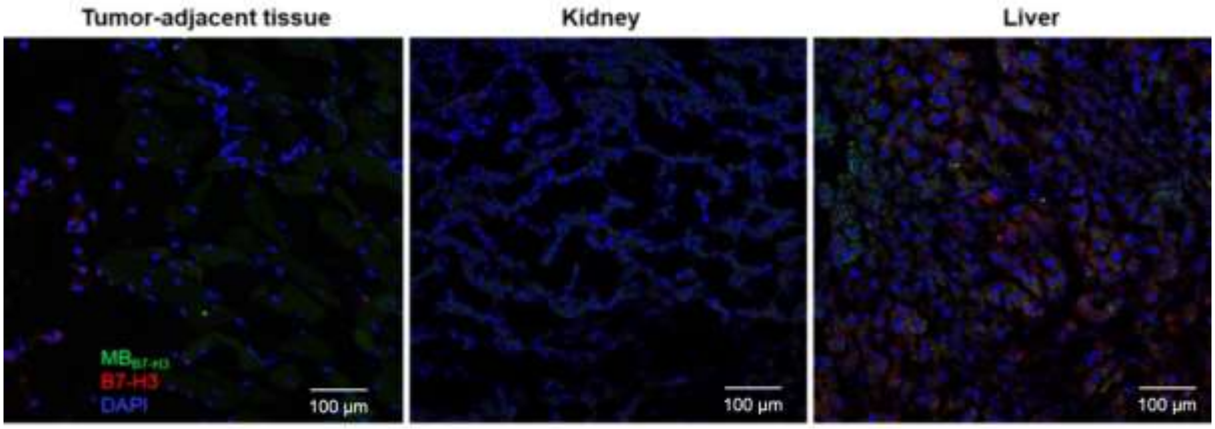

Figure S5. ***In vivo*** **MB$_{B7-H3}$ localization in normal tissues.** Representative composite fluorescence images from tumor-adjacent normal tissue (left), liver (middle), and kidney (right) showing signals from dye labeled MB$_{B7-H3}$ (green) and anti-B7-H3 Alexa Fluor 594 (red) immunostaining. Only liver shows diffuse signal for MBs suggesting their rapid hepatic clearance, while vascular B7-H3 is absent in all three tissue types. DAPI (blue) represents nuclear staining for cells.
